# Predicting the Anatomical Location of Neck of Femur Fractures in Osteoporotic Geriatric Indian Population

**DOI:** 10.5704/MOJ.2203.015

**Published:** 2022-03

**Authors:** MR Thirunthaiyan, K Mukherjee, TKR Prashanth, DR Kumar

**Affiliations:** Department of Orthopaedics, Sri Ramachandra Institute of Higher Education and Research, Chennai, India

**Keywords:** neck of femur, fracture, osteoporosis, Singh index

## Abstract

**Introduction:**

Neck of femur fractures are quite common fractures in the elderly. Though a lot is spoken about the various modes of management of these fractures across different age groups, hardly any literary support mentioning their distribution, location and pattern can be found. In this study, we aim to find whether the Singh index, as a marker of osteoporosis on digital radiographs, can predict the location of neck of femur fractures in the elderly population.

**Materials and methods:**

We accessed 556 fractured hip radiographs in our institution over the past 5 years (20152020) and correlated with the Singh index, as a marker of degree of osteoporosis, on pre-operative pelvis digital radiographs. Mid coronal CT cuts were also corroborated with the radiographic findings. A control group was set up and 361 radiographs were evaluated in the study group.

**Results:**

A total of 124 transcervical fractures (73%) were in Singh index 4, while 76 subcapital fractures (70%) were in Singh index 3. A total of 166 fractures (66%) were found in transcervical region in the age group of 60 to 80 years, while 80 fractures (74%) were in the subcapital region in patients above 80 years.

**Conclusion:**

We concluded that transcervical fractures were more common in patients with Singh index 4 (p<0.001) and subcapital more common in patients with Singh index 3(p<0.001). There was also a shift in location of the fractures from the transcervical region to the subcapital region with age above 80 years (p<0.001).

## Introduction

Hip fracture is a growing problem in the modern world and is a menace that is expected to worsen^1^. The devastating consequences of hip fractures for the patients and their families include an annual mortality rate of 30% with substantial impairment to the quality of life and independence^2^. A varied aetiology exists for neck of femur fractures ranging from the paediatric age group to the geriatric population. However, the advancing age, osteoporosis and metastatic lesions were found to be the major causes to weaken the bone tissues in elderly to such an extent that spontaneous fractures occur^3^. Localising the fracture and its type is important in young individuals, as it plays a crucial role in the decision making of its management and outcome. However, with the advancement of arthroplasty, it may seem quite irrelevant in the elderly population. Biomechanics at the proximal femur with respect to loading of the hip joint is also responsible for the varying fracture types^3^. Numerous classifications were proposed for fractures of the neck of femur in adults in the past, but all had limitations^4^. Femoral neck fractures were first classified as intra-capsular/extra-capsular and later classified as subcapital, mid-cervical, basal, and intertrochanteric or pertrochanteric types^5^. But there is no clear consensus on how to demarcate the femoral neck anatomically (subcapital/ transcervical/basicervical) and hence classifying them was difficult. Although there is enough literature to guide us with the management of these fractures across different age groups, there is hardly any literary material pertaining to the prediction of the possible location of these fractures.

**Fig. 1:**
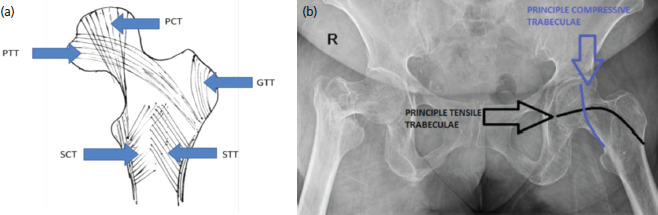
(a) Pictorial depiction of proximal femur trabecular pattern (b) Pelvis radiograph depicting the trabecular pattern of proximal femur.

Osteoporosis is a common feature in the elderly. Owing to the reduced bone mass, fragility fractures of the neck is a common incidence^6^. Bone mass is analysed by the Bone Mineral Densitometry (BMD), using an ultrasound or Dual Energy X-ray Absorptiometry (DEXA)^7^. Though considered a gold standard for detection of osteoporosis, DEXA cannot fully explain the risk of hip and vertebral fractures^8^. It has become increasingly apparent that the change in the trabecular pattern associated with a decrease in bone mass is more important than the amount of bone itself^9^. The trabecular pattern is a critical component, for the fracture risk prediction, that can be evaluated by radiographs and computed tomography (CT) scans^6^. Although proximal femur trabeculae are asymmetric, it follows a specific characteristic arrangement. There are five trabecular groups including- principal compressive trabeculae (PCT), principal tensile trabeculae (PTT), secondary compressive trabeculae (SCT), secondary tensile trabeculae (STT) and greater trochanter trabeculae (GTT)^6^. Based on these trabecular patterns observed in the proximal femur radiographs and on correlating it with the Singh index, osteoporosis can be graded (Fig. 1).

In our study, we aim to find the correlation between the location of the neck of femur fractures with the degree of osteoporosis and the advancing age using the Singh index. We hypothesise that patients with a Singh index 4 or above have a propensity to transcervical fractures, wherein patients with a poor bone stock (Singh index grade 3 or below) have a tendency for more proximal neck fractures (subcapital region). We further hypothesise that with advancing age and worsening osteoporosis, there is a proximal shift of location of the neck of femur fractures from basicervical to subcapital region.

## Materials and Methods

A single centre retrospective case control study spanned over five years (2015-2020) was designed. Radiographs of all the patients, aged 60 years or above, who sustained a femoral neck fracture were accessed from the PACS (picture archiving and communication system). Necessary ethics clearance was obtained from the institutional ethical committee. A total of 556 pelvic radiographs, CT scans and history sheets were scrutinised, and 361 patients were included in the study. Inclusion criteria were- history of slip and fall/ trivial trauma, age more than 60 years and ambulating prior to the injury. Patients with a history of high velocity impact injuries, history of contralateral hip surgeries, history of chronic renal failure, long standing steroid use, undergoing any treatment for osteoporosis, non-ambulant status prior to the injury, radiographs with arthritic changes at the hip, osteonecrotic changes and pathological fractures were excluded from the study. The selected patient’s femur head biopsies were further correlated to confirm osteopenia in the proximal femur and to rule out arthritis, osteonecrosis, or any evidence of pathological fractures.

**Fig. 2:**
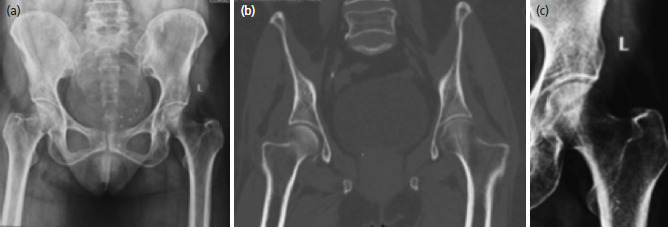
Case example, 80-year-old female, illustrating the trabecular pattern in proximal femur as evident on (a) pelvis radiograph (b) mid coronal CT cuts (c) magnified normal left hip for better visualisation of trabeculae. Hip graded as Singh index 3 based on trabecular pattern.

**Fig. 3:**
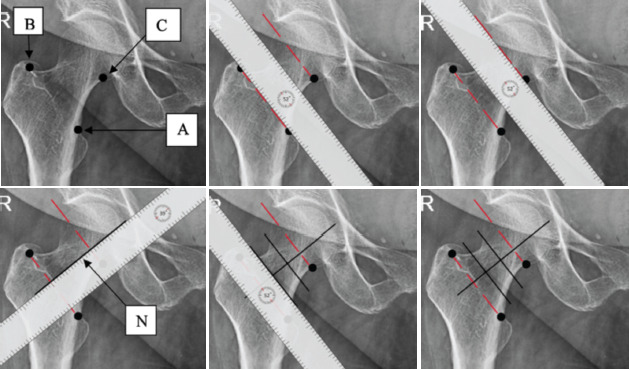
Illustration depicting method applied in dividing the neck of femur into three anatomical zones.

The pre-operative pelvis radiographs taken at the initial presentation were evaluated for osteoporosis using the Singh index (Fig. 2). Contralateral hip trabecular pattern and the proximal femur geometry were studied by three orthopaedic residents independently and the Singh index (Table I) was utilised to grade the degree of osteoporosis. All patients underwent CT pelvis and the trabecular arrangements in the proximal femur of the contralateral hip were studied by the principal investigator on the mid coronal cuts. The average reading of the three analysers was recorded (to the nearest whole number), after evaluation of the radiographs and ensuring appropriate blinding. Ipsilateral hip traction/internal rotation radiographs were then accessed to evaluate the location of the fractures in the neck. The neck region was divided into three equal zones and the fracture location was identified. The distal most line was drawn along the trochanteric ridge through two points. One point was at the upper border of the lesser trochanter (point A) and the other at the confluence of tip of the greater trochanter with the superior border of the neck (point B). Proximally, a point (point C) was taken at the medial neck- head junction. The proximal most line was drawn through the point C, parallel to the previous line joining points A and B. A perpendicular (line N) was drawn to these two lines passing through the fovea. Line N was then divided into 3 equal zones- upper one-third, middle one-third and lower one-third using Microsoft Office tools (Fig. 3). The anatomical zones– subcapital, transcervical and basicervical were representative of the upper one-third, middle one-third, and lower one-third part of the neck, respectively. The oblique fractures which started from one zone and ended in the other zone, were put into the zone where more than 50% of the fracture resided. The fractures were tabulated according to their anatomical zones after correlating with the Singh index. The patients were further divided into 3 age groups– 60 to 70 years, 70 to 80 years and above 80 years. The fracture patterns in the respective age groups were also studied.

**Table I:** Singh index grading and its corresponding radiological description on radiographs

Singh index	Description
6	All groups of trabeculae visible
5	PCT and PTT are attenuated, SCT not clearly demarcated
4	PTT decreased in number, STT not clearly demarcated
3	Break in continuity of PTT
2	Only PCT seen
1	Even PCT reduced in number

**Fig. 4:**
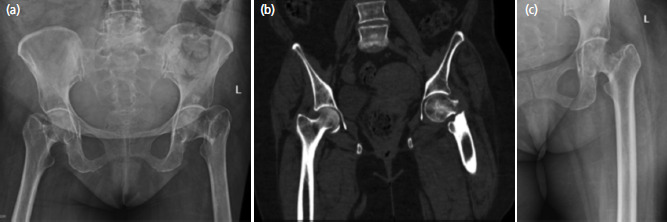
Case example from control group showing a 53-year-old female with Singh index 4 having a left hip basicervical fracture (a) pelvis radiograph AP (b) mid coronal CT image (c) traction internal rotation view of left hip.

A control group was setup with patients sustaining neck of femur fractures in the age group of 20 to 60 years. Similar inclusion and exclusion criteria were followed as in the study group. Three analysers independently evaluated the radiographs for Singh index, which were later graded by the principal investigator on CT films. Findings in the study and control groups were then closely correlated (Fig. 4).

## Results

A total of 361 fractures were evaluated. Table II depicts the demographic distribution of the patients. The study group consisted of 158 (43.8%) male patients and 203 (56.2%) female patients. Among these 179 (49.6%) were transcervical, 130 (36%) were subcapital and 52 (14.4%) were basicervical. The mean age was 74.88 years +/- 9.4, with patients in the age ranging from 60 to 110 years. Hypertension, diabetes, hyperlipidemia and hypothyroidism were the most common co-morbidities of the patients in the study group.

**Table II:** Demographic distribution of the patients

	Frequency (n)	Percentage (%)	Cumulative percentage
Gender			
Female	203	56.2	56.2
Male	158	43.8	100.0
Fracture Side			
Right	197	54.6	54.6
Left	164	45.4	100.0
Comorbids			
Hypertension	138	38.2	38.2
Diabetes	94	26.1	64.3
Hyperlipidemia	60	16.6	80.9
Hypothyroidism	43	11.9	92.8
Others	26	7.2	100.0
Fracture Location			
Basicervical	52	14.4	14.4
Transcervical	179	49.6	64.0
Subcapital	130	36	100.0

These fractures were spread over three different age groups– with 128 patients in 60 to 70 years group, 125 patients in 70-80 years group, and 108 patients in > 80 years group. The transcervical fractures accounted for more than 65% of the fractures in the age groups of 60 to 70 years and 70 to 80 years combined, while subcapital fractures were seen in 74% of the total fractures in elderly people with age above 80 years (Table III).

**Table III:** Age wise distribution of the fractures according to their anatomical location

Age	Basicervical	Transcervical	Subcapital
60-70 years	16	80	32
70-80 years	21	86	18
>80 years	15	13	80

Table IV shows the patient distribution according to the Singh index. The selected patients were evaluated for osteoporosis according to the Singh index ranging from 5 to 1. Not a single case was found to have an index of 6. The Singh index 5 group had 12 basicervical, 22 transcervical and 8 subcapital fractures. A total of 124 transcervical patients were found in the Singh index 4 group with 29 and 18 patients in subcapital and basicervical categories respectively. Singh index 3 had 76 patients grouped into subcapital, 20 as transcervical and 12 as basicervical fractures.

**Table IV:** Distribution of the fractures according to Singh index

Singh Index	Basicervical	Transcervical	Subcapital
5	12	22	8
4	18	124	29
3	12	20	76
2	10	12	16
1	0	1	1

Control group had only 43 patients, with 31 being female and 12 as male patients. The mean age was 52.16 years +/- 7.12, with age ranging from of 44 to 58 years. On evaluating the radiographs and CT scans, all these patients were in Singh index 6, 5 and 4. Only one patient had a subcapital location of neck of femur fracture from the entire control group (Table V).

**Table V:** Control group demographics

Parameters	Values
Gender	
Male	12
Female	31
Mean Age	52.16 years +/- 7.12
Singh index	
6	10
5	29
4	4
3	0
2	0
1	0
Location of fracture	
Subcapital	1
Transcervical	28
Basicervical	14

IBM SPSS statistical software [version 21] was used for data analysis. A simple descriptive statistical analysis and Chi-square test was used to plot the frequencies, percentage, and prevalence of the demographic data. Cross tabulations were done between the Singh index and the individual fracture locations (basicervical, transcervical and subcapital). Cross-tabulation between the different age groups and the anatomical location of the fractures in the femoral neck were also studied. Statistical analysis was done to find any correlation between sex distribution, co-morbidities, and location of these fractures as well.

## Discussion

Neck of femur fractures are common in elderly population with a trivial fall. Age related osteoporosis is often blamed as the leading cause behind it. While many studies have been done in the past formulating the treatment strategies, a void to explain the exact biomechanics and the pattern of these fractures in osteoporotic geriatric population still exists. The primary objective of our study was to find whether any pattern exists and if we can predict the location of these fractures correlating with the degree of osteoporosis.

A study by Augat *et al* on the biomechanics of femoral neck fractures stated sideways impact on the greater trochanter resulting in compressive loading of the femoral neck as the most common mechanism of these fractures in the old age^3^. Apart from the bone mineral density at the femoral neck, other factors like bone geometry, cortical thickness, and cortical bone density are important factors affecting the risk of neck fractures^10^. Hip axis length, neck length and cross-sectional area of the neck also influence the risk of these fractures^10,11^.

In 1970, Singh *et al* described a simple method to estimate the level of bone mineral density on radiographs^12^. By morphometric analysis and correlation to histologic findings, the author suggested that a loss in bone mineral density resulted in changes in trabecular pattern of the proximal femur on plain anterior-posterior pelvic radiographs^13^. A scale of one to six was designed based on these trabecular patterns, with one being highly osteoporotic and six being the least. Superiority of the recent digital radiographs have made the judgement of these trabecular patterns easier^14,15^, but it is quite subjective and has high inter observer variability. Watcher *et al*^16^ and Bes *et al*^17^ in their studies found a good correlation between the bone mineral density and Singh index. Their study recorded good inter-rater reliability between these two parameters based on the results of three observers. We have utilised the same scoring system to evaluate the degree of osteoporosis based on the mean scoring of three independent observers.

Localising these neck of femur fractures needs a proper classification. A fracture classification is proposed either for taxonomical purpose, characterisation, guiding intervention or predicting the outcomes of intervention^4^. Any useful system should consider the severity of the injury, be the guide to treatment and facilitate the comparison of result^18^. The anatomical classification dividing the neck fractures into subcapital, transcervical and basicervical zones, has no clear demarcation of the regions. This forced us to categorise the neck region into three equal zones and label them as upper one-third, middle one-third and lower one-third which almost resembles rather overlaps the traditional anatomically classified zones. The fractures were then plotted according to the degree of osteoporosis and the Singh index, and it showed a pattern.

Osteoporosis is best graded in modern medicine by the BMD, which can be evaluated using DEXA scans. However, Tristan *et al*^19^ in his study on age related cortical and trabecular bone changes at femoral head stated that BMD evaluation is over generalising the osteoporosis at proximal femur and that the trabecular structure in the proximal femur is more important than the amount of bone. Lu *et al*^6^ studied the trabecular distribution of proximal femur in patients with fragility fractures and stated that the BMD values vary across fractures and non-fractures. Their study stressed upon the reliability of the trabecular pattern analysis that determines the hip fracture susceptibility. The authors further stated that hip fractures are usually around the inter- trochanteric region and subcapital region where there is shortage of cortex. The study concluded that femoral neck fractures have a close relationship with the degeneration of compressive and tensile trabeculae and hence the risk of hip fractures can be evaluated by the study of the proximal femur trabeculae alone. Our study evaluated the proximal hip trabecular pattern on the digital radiographs and utilised the Singh index to grade the osteoporosis. Although multiple studies state CT as a better indicator for BMD, there are a lot of minute adjustments to be made for such reliable results. Lu *et al*^6^ mentioned neutral position of limbs, 1mm cut interval and a multi detector computed tomography (MDCT) to be preferred over the quantitative computed tomography (QCT) for best results. QCT only measures the quantity of trabeculae and cannot distinguish its distribution in the proximal femur. In our study, the principal investigator did an evaluation of all the cases and controls by MDCT scans which were then correlated with the radiographic evaluation by the independent analysers. A good inter and intra-observer reliability was noted (p<0.05), thus proving that digital radiographs are equally sensitive for analysis of femoral trabeculae (Table VI).

**Table VI:** Inter-observer reliability of the analysers

	Investigations	Singh index 6	Singh index 5	Singh index 4	Singh index 3	Singh index 2	Singh index 1	P value
A 1	Radiographs	--	33	168	50	40	1	.013
A 2	Radiographs	--	29	172	46	32	2	.021
A 3	Radiographs	--	30	173	48	42	2	.033
P.I	CT scans	--	31	171	49	37	2	--
Mean		--	31	171	48	38	2	

Abbreviations: A 1- Analyser 1, A 2- Analyser 2, A 3- Analyser, P.I- Principal investigator

The functional adaptation of trabecular bone is a well-established phenomenon, which adapts to the external loading conditions, a condition better explained by Wolff’s law^19^. Due to aging, trabeculae in the tensile regions might decrease, but those along the principal compressive axis are largely maintained and this often makes the hip susceptible to fracture on sideway falls^19^. Tristan *et al*^19^ studied the age-related changes in cortical and trabecular bone in old age of 70 to 93 years. A significant decrease in global trabecular bone density (38.1%) and cortical thickness (13%) was noted from the 9th to 10th decade of life. This loss of trabecular bone was noted in both high stress and low stress areas of the femoral neck. The study concluded that the role of functional adaptation in maintaining the structural integrity of bone in old age is questionable even in ambulating patients. Our study showed similar results, where all the ambulating patients above the age of 80 years showed significant decrease in compressive trabeculae in the femoral neck and recorded poor Singh index score.

**Fig. 5:**
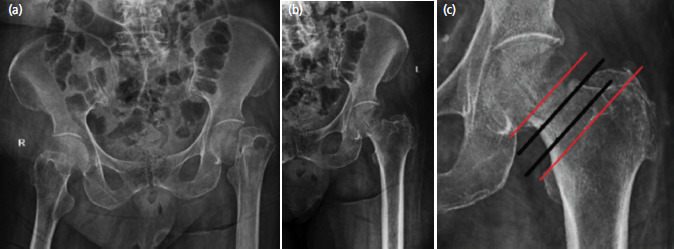
(a, b, c) Radiographs depicting 82-year-old female with Singh index grading 3 who sustained a subcapital fracture.

**Fig. 6:**
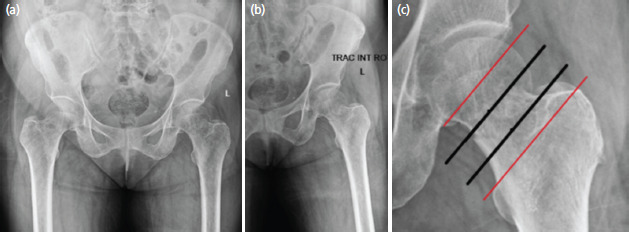
(a, b, c) Radiographs depicting 66-year-old female with Singh index grading 4 who sustained a transcervical fracture.

In our study, majority of the patients were represented by Singh index 4 (47%) and 3 (30%). In these two groups, we found that majority of the fractures in Singh index 4 were transcervical (73%) with a p value <0.001 (Fig. 5) and patients with Singh index 3 had subcapital fractures (70%) with a p value <0.001 (Table VII). A shift in the location of the fractures was seen with age- transcervical fractures being the common zone in 60 to 70 years (p<0.001) and 70 to 80 years population (p<0.01) and subcapital being the most affected zone (Fig. 6) in population aged above 80 years (p<0.001). This shift further corroborated with our finding with the Singh index and location of the fractures. Females in post-menopausal age group are more susceptible to hip fractures20, which made us record higher female fractures (56.2%) as compared to males (43.8%) but no significant differences in location of the fractures with gender was noted in our study. Co-morbidities of the patient also did not play any significant factor in determining the site of neck of femur fractures.

**Table VII:** Descriptive statistics with crosstab between Singh index and fracture location

Fracture Location	Singh index (S.I)				Chi- square Test (P value)
	S.I Grade	Fracture present	Fracture absent	Total	
Transcervical	1	1	1	2	.000
	2	13	26	39	
	3	19	88	107	
	4	125	47	172	
	5	21	20	41	
Basicervical	1	0	2	2	.005
	2	10	29	39	
	3	12	95	107	
	4	18	154	172	
	5	12	29	41	
Subcapital	1	1	1	2	.000
	2	16	23	39	
	3	76	31	107	
	4	29	143	172	
	5	8	33	41	

We acknowledge certain limitations in our study. First, Singh index as a parameter to measure the porosity of bone is questionable. Although we have literature suggesting Singh index as a cheap, readily accessible, and available tool to indirectly measure the bone mineral density, we also have enough literature stating how unreliable it is in grading the severity of osteoporosis. We have evaluated the contralateral hip for osteoporosis and thus assumed the trabecular patterns to be almost similar bilaterally. Shankar *et al*^21^ who studied the trabecular changes of proximal femur in postmenopausal women, did not find any significant difference of the proximal femur trabeculae in the bilateral hips. Second, localising the fractures on the neck of femur based on the anatomical classification is difficult. This classification has not clearly demarcated the extent of these zones. Dividing the entire neck into three equal zones is over magnifying the subcapital and basicervical fractures and under reporting the transcervical ones. But the proximal shift of fractures from distally located basicervical area to more proximal subcapital area with advancing age and worsening osteoporosis, remains clear and vivid and do not affect our study findings.

## Conclusion

We found that most of the femoral neck fractures in the geriatric population were in the middle one-third of the neck, representative of the transcervical region. The transcervical fractures were more common in patients with Singh index 4 and subcapital fractures in patients with Singh index 3. We further concluded that there was a definite proximal shift of these fractures from transcervical to subcapital region with age over 80 years.

Predicting the location of neck of femur fractures is multi factorial. Assessing the grade of osteoporosis and correlating with the age gives an idea about their location and pattern of their distribution, but there are other risk factors associated which also need to be evaluated. Predicting the location of the neck of femur fractures might seem irrelevant in the elderly, as majority of them are treated by hip arthroplasty, but makes one curious as to why there is a marked difference to the location of these fractures with the advancing age and worsening osteoporosis.
